# Advances in Infection Prevention for Pediatric and Neonatal Populations: Classification of Methods and the Emerging Superiority of Ultraviolet-C (UV-C) Technologies

**DOI:** 10.7759/cureus.89578

**Published:** 2025-08-07

**Authors:** Mitchell K Ng, Michael A Mont, Peter M Bonutti

**Affiliations:** 1 Orthopaedic Surgery, Maimonides Medical Center, Brooklyn, USA; 2 Orthopaedic Surgery, LifeBridge Health, Baltimore, USA; 3 Orthopaedic Surgery, Sarah Bush Lincoln Bonutti Clinic, Effingham, USA

**Keywords:** chemical disinfectants, infection prevention, neonatal, pediatric patients, uv-c emitters

## Abstract

Healthcare-associated infections (HAIs) continue to pose major risks to pediatric and neonatal patients, whose immature immune systems and unique vulnerabilities demand tailored infection prevention strategies. Traditional methods, including chemical disinfectants, procedural protocols, and physical hygiene measures, have contributed to reductions in HAIs but remain limited by human error, environmental toxicity, and the rise of antimicrobial resistance. Advances in disinfection technologies, particularly ultraviolet-C (UV-C) systems, offer promising new avenues for safer, more effective pathogen control. This review systematically classifies infection prevention methods into chemical, physical, procedural, and emerging technology categories, emphasizing their respective strengths and limitations. Special attention is given to UVCeed (UVCeed Inc., Effingham, Illinois, United States), a next-generation UV-C disinfection device that delivers rapid, targeted, and chemical-free decontamination with minimal reliance on human compliance. UVCeed's integration into pediatric infection control protocols represents a major advance, addressing critical gaps left by conventional methods. By synthesizing current knowledge and highlighting future directions, this article aims to guide clinicians, infection control specialists, and healthcare administrators toward more comprehensive, technology-enhanced approaches to safeguarding pediatric patients.

## Introduction and background

Healthcare-associated infections (HAIs) remain a major concern in pediatric healthcare, particularly affecting neonates, infants, and immunocompromised children [1]. These populations are especially vulnerable due to their underdeveloped immune systems, delicate skin barriers, and frequent exposure to invasive procedures such as catheter placements and intubations [2]. According to the Centers for Disease Control and Prevention (CDC), approximately one in 31 hospitalized patients in the United States has at least one HAI on any given day [3]. In neonates, sepsis is a leading cause of morbidity and mortality, with incidence rates ranging from one to 10 cases per 1,000 live births globally [4].

Despite notable advancements in antimicrobial therapies and infection control protocols, HAIs persist across neonatal intensive care units (NICUs), pediatric intensive care units (PICUs), and general pediatric wards, highlighting the need for improved strategies. Infection control is especially critical in pediatric surgical specialties, including orthopaedics, where postoperative infections such as surgical site infections (SSIs) can have devastating long-term consequences on bone growth and functional outcomes [5]. Pediatric orthopaedic procedures, often involving implanted devices, are particularly susceptible to infection, making stringent prevention protocols essential to improving surgical success rates and minimizing complications [6].

Traditional infection prevention methods have primarily relied on chemical disinfectants, adherence to procedural protocols, and basic hygiene practices such as handwashing [7]. While these approaches have led to improvements, their effectiveness is often limited by factors like human error, inconsistent compliance, and environmental contamination [7]. Moreover, the overuse of chemical agents raises concerns about toxicity, environmental impact, and the emergence of resistant microbial strains [8,9]. In response to these challenges, there has been growing interest in innovative technologies that offer consistent, high-level disinfection without the associated risks of chemical methods [10,11]. Ultraviolet-C (UV-C) light technologies have gained attention for their ability to rapidly and effectively inactivate a wide range of pathogens [12,13]. The global UV disinfection equipment market is projected to grow from USD 4.31 billion in 2023 to USD 13.86 billion by 2031, recording a compound annual growth rate (CAGR) of 15.7% during the forecast period [14]. Devices like UVCeed (UVCeed Inc., Effingham, Illinois, United States) represent important advancements in infection prevention, offering enhanced efficacy, user safety, and operational efficiency [15-17].​

This review aims to systematically classify current infection prevention methods used in pediatric care, critically evaluate their strengths and limitations, and highlight the emerging superiority of UV-C technologies, with a specific focus on UVCeed. By providing a comprehensive overview, this article seeks to inform clinicians, infection control specialists, and healthcare administrators about the evolving landscape of pediatric infection prevention and the promising role of advanced disinfection technologies.

## Review

Unique considerations for pediatric patients

The prevention of infections in pediatric and neonatal patients demands careful attention to their distinctive physiological and clinical vulnerabilities [17]. Neonates, especially those born prematurely, face profound risks from infection due to immature immune responses and fragile integumentary barriers [18]. Their skin, much thinner and more permeable than that of adults, allows easier microbial invasion and chemical absorption [19]. According to the World Health Organization, neonatal infections are responsible for more than 550,000 deaths annually worldwide [20]. In the United States alone, the incidence of neonatal sepsis is approximately one to two cases per 1,000 live births [21]. Routine interventions such as central lines, endotracheal tubes, and urinary catheters, while necessary for survival, act as portals for nosocomial pathogens. Compounded by immature hepatic and renal systems, which reduce the ability to clear toxins, neonates are at substantially greater risk for systemic toxicity from disinfectants and antimicrobial agents [22]. Every aspect of infection control, from the type of disinfectant used to the method of application, must be precisely adapted to minimize harm while maximizing protection.

Behavioral and environmental factors further complicate infection control in these populations. Infants and young children, who lack hygiene awareness, frequently contaminate themselves and their surroundings by touching surfaces, medical devices, and caregivers [2,3]. Pediatric hospitals are typically high-contact environments with a greater number of caregiver interactions compared to adult settings. A 2023 study noted that the HAI rate among hospitalized children is approximately 3.9%, with device-associated infections accounting for a major proportion [23]. Family-centered care, while crucial for emotional and developmental support, introduces additional risks, as parental presence increases the volume of room traffic and potential environmental contamination. These realities necessitate the integration of infection prevention strategies that are robust, sustainable, and minimally reliant on human compliance. Advanced technologies such as UVCeed [15], which offer rapid, targeted, and chemical-free surface disinfection, are particularly well-suited for the pediatric environment, providing an indispensable adjunct to traditional methods and markedly enhancing the overall safety net for the most vulnerable patients.

Known dangers of chemical disinfectants

Chemical disinfectants pose serious systemic risks in pediatric populations due to absorption through the skin, respiratory tract, and oral mucosa, with the potential to cross the blood-brain barrier (BBB) [11]. In addition to this risk, pediatric patients often struggle to follow protocols for conventional disinfection or prevention strategies, making them an especially vulnerable patient population. Increasing evidence highlights the biological vulnerability of infants and young children to chemical disinfectants, particularly quaternary ammonium compounds (QACs), which are ubiquitous in healthcare environments [9,10]. Notably, many physicians do not wholly understand the risks and complications of chemical disinfectants, such as application time, residual chemicals, required rinsing of hands and surfaces after use, and the risk of spreading harmful agents through oral, nasal, inhalational, or dermal absorption [9,11].

A 2022 study found QAC residues in 72% of breast milk samples tested, with concentrations of C14-benzalkonium chloride (C14-BAC) reaching a median of 0.45 ng/mL and total QAC levels up to 7.4 ng/mL, posing an exposure risk of up to 750 ng/kg/day in nursing infants [24]. These findings are especially alarming given the immature skin barrier and reduced hepatic and renal clearance in neonates, which amplify systemic absorption and toxic accumulation [25]. Experimental animal models have demonstrated that bis-quaternary ammonium compounds, such as hexamethonium bromide [26], can traverse the placental barrier, directly exposing the developing fetus to compounds not intended for systemic absorption and resulting in potential neural defects [27]. These findings implicate routine disinfection practices as a previously underestimated pathway of neonatal and perinatal toxicant exposure.

A potential limitation in pediatric infection control arises when disinfecting items or surfaces that directly contact sensitive tissues (e.g., mouth, eyes, open wounds). In such cases, chemical disinfectants carry risks including toxic absorption, mucosal irritation, and inhalational exposure. Compounding the issue is the inability to visually identify treated versus untreated areas, as most chemical agents are colorless and leave behind residues that are rarely removed, despite labeling instructions. While single-use items may reduce some of these hazards, they are expensive, generate waste, and offer inconsistent efficacy. Another underrecognized issue in current protocols is that standard protective equipment, such as gloves, masks, and gowns, primarily safeguards the healthcare provider rather than the patient. Current systems prioritize provider safety, yet fail to address the persistent microbial threats present in the patient's immediate environment.

An area of growing concern is the potential neurodevelopmental impact of early-life exposure to QACs and other disinfectants. In vitro research from Case Western Reserve University has shown that QACs from countless household items disrupt oligodendrocyte maturation and function, critical for central nervous system myelination, raising red flags for long-term cognitive outcomes and associated with multiple sclerosis and autism spectrum disorders [28]. Animal studies corroborate this risk, with perinatal QAC exposure linked to impaired memory, increased locomotor activity, and behaviors consistent with attention-deficit hyperactivity disorder (ADHD) phenotypes [29]. These neurological changes likely stem from disrupted cholinergic signaling and oxidative stress pathways during brain development [28]. Such data not only suggests a mechanistic basis for the observed rise in learning and ADHD disorders but also reinforces the urgency of reevaluating disinfectant use in environments housing infants and children. As the developmental brain proves exquisitely sensitive to environmental toxicants, healthcare protocols must be scrutinized to prevent inadvertent harm from the very agents meant to protect.

Classification of infection prevention methods

Infection prevention methods can be broadly classified into four categories: chemical disinfection, physical disinfection, procedural controls, and emerging technologies [30]. Table 1 summarizes key methods, examples within each category, and their primary applications.

**Table 1:** Classification of pediatric infection prevention methods Table Credits: Mitchell K. Ng References: [1-3,10,12,27-30]

Type	Example	Application
Chemical disinfection	Chlorhexidine gluconate (CHG)	Skin and line antisepsis
	Povidone-iodine	Preoperative skin preparation; cord care
	Alcohols (isopropyl, ethanol)	Hand hygiene; surface cleaning
	Quaternary ammonium compounds (QACs)	Surface disinfection
	Sodium hypochlorite (bleach)	Environmental disinfection
	Hydrogen peroxide	Surface and equipment disinfection
Physical disinfection	Hand hygiene	Alcohol-based rubs, soap, and water
	Barrier protection	Gloves, gowns, masks
	HEPA filtration	Airborne pathogen removal in critical care
Procedural controls	Central line insertion bundles	Sterile insertion and maintenance protocols
	Surgical site infection (SSI) bundles	Preoperative antisepsis; sterile techniques
	Isolation precautions	Contact, droplet, and airborne precautions
	Antimicrobial stewardship programs	Rational antibiotic use
Emerging technologies	Ultraviolet-C (UV-C) disinfection	Surface and air sterilization
	UVCeed	Targeted UV-C device for surface disinfection
	Antimicrobial surface coatings	Copper/nano-silver materials for passive disinfection
	Robotics and automated disinfection	Robotic UV-C disinfection and compliance tracking

Chemical disinfection

Chemical disinfection has long been a central component of infection prevention efforts in pediatric and neonatal care settings [30]. Agents such as chlorhexidine gluconate (CHG) and povidone-iodine are widely utilized due to their reliable broad-spectrum antimicrobial properties, rapid onset of action, and established safety profiles when used appropriately [31]. These disinfectants are particularly effective for preoperative skin preparation and catheter site maintenance, offering healthcare providers tools that are both familiar and extensively validated across clinical contexts. Their advantages include ease of use, widespread availability, and the ability to deliver consistent results when protocols are followed meticulously [31].

However, there are important limitations to chemical disinfection, especially in vulnerable neonatal populations. The immature skin of neonates increases the likelihood of systemic absorption, leading to potential toxicities [22]. Repeated exposure to chemical agents can disrupt normal skin flora, impair wound healing, and contribute to antimicrobial resistance. Additionally, environmental concerns related to chemical residues, coupled with the risk of human error in dilution or application, highlight the need for rigorous oversight [32]. As a result, while chemical disinfectants remain foundational, they must be used thoughtfully, often in combination with newer technologies that can mitigate these risks.

Physical disinfection

Physical disinfection methods, encompassing practices such as hand hygiene and barrier protection, are critical pillars of infection prevention in pediatric healthcare [33]. Proper and consistent hand hygiene remains the single most effective measure for reducing HAIs, and the use of gloves, gowns, and masks serves to further minimize direct pathogen transmission [34]. The advantages of these strategies are clear: they are non-toxic, cost-effective, environmentally sustainable, and generally well-tolerated across all patient populations [34]. In critical care settings like NICUs, HEPA filtration systems add another layer of protection by removing airborne contaminants, contributing importantly to safer clinical environments [35].

Nevertheless, the effectiveness of physical disinfection is heavily reliant on human behavior and compliance. Breakdowns in hand hygiene protocols or inconsistent use of personal protective equipment (PPE) can critically undermine infection control efforts. Additionally, environmental technologies such as HEPA systems, while beneficial, require ongoing maintenance and operational investment, potentially limiting access in resource-constrained settings [35]. These limitations underscore the importance of supplementing manual physical disinfection with automated and technology-driven interventions that can help ensure consistency and reliability.

Procedural controls

Procedural controls offer structured, standardized methods for reducing infection risks by embedding best practices into daily clinical routines. Central line insertion bundles, SSI prevention protocols, and antimicrobial stewardship initiatives have collectively led to major improvements in patient safety outcomes [36]. These controls enhance uniformity, minimize human error, and provide measurable frameworks for continuous quality improvement. Their adoption has fostered a stronger culture of safety within pediatric healthcare, aligning multidisciplinary teams around shared goals of infection reduction [37].

However, these benefits are not without challenges. Procedural adherence requires constant education, diligent monitoring, and a sustained institutional commitment [36]. Staff fatigue, high turnover rates, and fluctuating resources can erode compliance over time. Isolation protocols, while necessary for controlling infectious outbreaks, can also place emotional strain on young patients and their families [38]. Therefore, procedural controls are most effective when reinforced by technological innovations such as UV-C disinfection systems, which provide a reliable safety net that does not waver with human variability [39].

Emerging technologies

Emerging technologies, particularly UV-C disinfection platforms like UVCeed, are redefining the future of infection prevention in pediatric and neonatal care. UV-C light systems provide rapid, high-level microbial inactivation without the drawbacks associated with chemical exposure [12,40]. The UVCeed distinguishes itself by offering portable, targeted, and user-friendly solutions that integrate seamlessly into existing clinical workflows. Key advantages include reduced reliance on manual labor, minimized risk of human error, chemical-free operation, and built-in compliance tracking to verify proper use [12].

Despite these substantial strengths, UV-C technologies are not without minor limitations [13]. The initial financial investment can be high, and UV-C devices must be used according to safety protocols to prevent unintended exposure [12]. Furthermore, UV-C disinfection must complement, rather than replace, manual cleaning for organic material removal. Nevertheless, the net benefits, including greater operational efficiency, lower HAI rates, and enhanced patient safety, make UV-C systems like UVCeed an essential addition to any modern infection prevention strategy [13,41,42]. The UVCeed integrates artificial intelligence, cameras, sensors, and computer systems to deliver precise UV-C dosing, confirm which areas have been disinfected, and ensure safe, consistent application tailored to each environment. To this end, it can serve as an efficient, environmentally safe adjunct to traditional disinfection methods, eliminating the need for chemical residues or disposable waste while providing an added layer of protection.

Their role in creating safer environments for vulnerable pediatric populations is only expected to grow as healthcare systems increasingly prioritize technological integration [43].

Comparative evaluation of infection prevention methods

When assessing various infection prevention strategies, it becomes evident that each method carries distinct benefits and inherent limitations. Chemical disinfection has long served as a mainstay, prized for its broad antimicrobial coverage, cost accessibility, and predictable performance [30]. Yet, its effectiveness is closely tied to meticulous application techniques, and concerns remain regarding chemical exposure risks and environmental impact, prompting a need for supplementary solutions [44]. Physical measures, such as rigorous hand hygiene and use of protective barriers, are both economical and highly effective when applied consistently. However, their success heavily depends on human behavior, and lapses in adherence can severely weaken protective efforts.

Structured procedural controls, including standardized bundles and antimicrobial stewardship initiatives, have fundamentally strengthened patient safety culture, fostering consistent, evidence-based practices [45]. Nevertheless, these frameworks require persistent investments in training, oversight, and administrative support, and their success can waver in fast-paced, resource-limited environments. While isolation and stewardship are cornerstones of infection prevention, they must be continually reinforced to maintain their effectiveness [46].

Emerging technologies, and notably UV-C disinfection systems such as UVCeed, address many of the vulnerabilities that traditional methods expose [41,47]. Chemical disinfectants, while effective, lack visual confirmation of treated surfaces and leave behind invisible and potentially hazardous residue, especially in sensitive pediatric settings. In contrast, UVCeed enables visible, verifiable disinfection without potential byproducts requiring removal or environmental hazards. Integrated artificial intelligence and visual tracking systems confirm what has or has not been treated, providing next-generation transparency and safety. The UV-C disinfection provides a uniform, high-level microbial kill without reliance on chemicals or perfect human execution. The UVCeed, specifically, advances disinfection protocols by delivering mobile, precision-targeted pathogen eradication with built-in safety mechanisms and compliance tracking [15]. Its rapid disinfection cycles allow healthcare teams to maintain stringent infection control without disrupting clinical workflows. Early studies indicate that supplementing manual cleaning with UVCeed dramatically improves bioburden reduction, particularly in vulnerable pediatric settings [48].

Ultimately, no single method is sufficient in isolation (Table 2). The optimal infection prevention strategy integrates chemical, physical, and procedural methods alongside emerging technologies like UV-C disinfection. UVCeed exemplifies how new tools can fill critical gaps left by traditional approaches, offering scalable, effective, and technologically advanced protection [49]. The future of pediatric infection control lies in comprehensive, multimodal strategies where innovative solutions and proven practices work synergistically to safeguard the most vulnerable patients.

**Table 2:** Summary of pros and cons by method Table Credits: Mitchell K. Ng References: [1-3,30-32,35]

Method	Advantages	Disadvantages
Chemical disinfection	Rapid bactericidal activity, widely available, standardized protocols, effective against a broad range of pathogens	Potential skin irritation; systemic toxicity risks; environmental chemical burden; potential to foster microbial resistance
Physical disinfection	Low-cost, minimal environmental impact, effective with consistent application, safe for all patient populations	Highly dependent on human compliance; variable effectiveness; requires continuous education and monitoring
Procedural controls	Promotes standardization, reduces human error, fosters a culture of safety, supports sustainable infection control practices	Resource-intensive; requires ongoing staff training and monitoring; vulnerable to compliance fatigue over time
Emerging technologies (UV-C, UVCeed)	High-level microbial eradication, consistent, reproducible disinfection; chemical-free operation; mobile and targeted, rapid cycle times; minimal reliance on human compliance, integrated compliance tracking, seamless integration into workflows. UVCeed provides unmatched precision, superior safety protocols, and operational efficiency, clearly surpassing traditional methods	Initial investment costs; requires safe handling practices for UV-C exposure; needs integration into manual cleaning workflows for optimal performance

Future directions

The future of infection prevention in pediatric and neonatal care is increasingly shaped by technological advancements, with UV-C disinfection emerging as a pivotal tool [43,50]. The global healthcare UV disinfection equipment market is projected to grow at a CAGR of 6% from 2024 to 2031, reflecting the rising demand for effective pathogen elimination in healthcare settings [51]. This growth is driven by the need for enhanced cleaning and disinfection of environmental surfaces, which are essential elements of infection prevention programs.​

In PICUs and NICUs, the implementation of UV-C disinfection has shown promising results. For instance, the installation of UV-C air disinfection devices in intensive care units (ICUs) led to a 42% reduction in microbiological air contamination, markedly improving the hospital environment's overall cleanliness [52]. This reduction in airborne pathogens was associated with a marked decline in catheter-related infections, highlighting the effectiveness of UV-C technology in reducing HAIs [52].

As UV-C technology continues to evolve, its integration into pediatric and neonatal infection prevention protocols is anticipated to become more widespread. The development of mobile, automated UV-C disinfection systems allows for rapid and consistent decontamination of patient rooms and equipment [53,54], minimizing the reliance on manual cleaning and reducing the risk of human error. These advancements not only enhance patient safety but also contribute to operational efficiency within healthcare facilities.

## Conclusions

Effective infection prevention in pediatric and neonatal care demands approaches that go beyond traditional chemical and procedural methods. While longstanding strategies have provided a crucial foundation, their limitations, especially those related to compliance variability, chemical toxicity, and antimicrobial resistance, highlight the need for innovative solutions. UV-C disinfection technologies, and particularly platforms like UVCeed, have emerged as powerful adjuncts, offering rapid, consistent, and safe pathogen eradication with minimal reliance on human behavior. Integrating UV-C disinfection into pediatric infection control frameworks has the potential to transform standards of care, tremendously lowering HAI rates and improving patient outcomes. As pediatric healthcare continues to evolve, embracing next-generation disinfection methods alongside established best practices will be key to protecting the most vulnerable patients and advancing the future of infection prevention.
